# SNiPer: Improved SNP genotype calling for Affymetrix 10K GeneChip microarray data

**DOI:** 10.1186/1471-2164-6-149

**Published:** 2005-10-31

**Authors:** Matthew J Huentelman, David W Craig, Albert D Shieh, Jason J Corneveaux, Diane Hu-Lince, John V Pearson, Dietrich A Stephan

**Affiliations:** 1 Neurogenomics Division, The Translational Genomics Research Institute (TGen) Phoenix, Arizona 85004, USA

## Abstract

**Background:**

High throughput microarray-based single nucleotide polymorphism (SNP) genotyping has revolutionized the way genome-wide linkage scans and association analyses are performed. One of the key features of the array-based GeneChip^® ^Mapping 10K Array from Affymetrix is the automated SNP calling algorithm. The Affymetrix algorithm was trained on a database of ethnically diverse DNA samples to create SNP call zones that are used as static models to make genotype calls for experimental data. We describe here the implementation of clustering algorithms on large training datasets resulting in improved SNP call rates on the 10K GeneChip.

**Results:**

A database of 948 individuals genotyped on the GeneChip^® ^Mapping 10K 2.0 Array was used to identify 822 SNPs that were called consistently less than 75% of the time. These SNPs represent on average 8.25% of the total SNPs on each chromosome with chromosome 19, the most gene-rich chromosome, containing the highest proportion of poor performers (18.7%). To remedy this, we created SNiPer, a new application which uses two clustering algorithms to yield increased call rates and equivalent concordance to Affymetrix called genotypes. We include a training set for these algorithms based on individual genotypes for 705 samples. SNiPer has the capability to be retrained for lab-specific training sets. SNiPer is freely available for download at .

**Conclusion:**

The correct calling of poor performing SNPs may prove to be key in future linkage studies performed on the 10K GeneChip. It would prove particularly invaluable for those diseases that map to chromosome 19, known to contain a high proportion of poorly performing SNPs. Our results illustrate that SNiPer can be used to increase call rates on the 10K GeneChip^® ^without sacrificing accuracy, thereby increasing the amount of valid data generated.

## Background

Single nucleotide polymorphisms (SNPs) are fast becoming the markers of choice for genome-wide linkage scans, loss of heterozygosity (LOH), comparative genomic hybridization (CGH) and whole-genome association studies [1]. This is due to the existence of high throughput technologies like the GeneChip^® ^Human Mapping Array from Affymetrix coupled with the abundant and uniform distribution of SNPs throughout the human genome [2-6]. The GeneChip^® ^Mapping Array relies on the hybridization of biotin-tagged fragments of SNP-containing DNA to complementary DNA oligomers chemically tiled on a silicon wafer in order to genotype 10,204 SNPs with a mean inter-marker spacing of 258 Kb [7]. The assay utilizes a relatively minor amount of genomic DNA (250 ng) and a series of reactions called fragment selection by PCR (FSP). The FSP reactions involve an Xba I restriction enzyme digest of genomic DNA followed by a universal adaptor ligation step and then PCR using parameters designed to selectively amplify DNA less than 1 Kb in size. After purification, the PCR products are digested to a size of ~50 bp with DNase I, end-labeled with biotin, and hybridized to the microarray wafer.

Successful hybridizations are detected fluorescently using a streptavidin-phycoerythrin conjugated molecule and an antibody-mediated signal amplification technique. Each SNP is interrogated in both the sense and antisense direction by multiple "quartets" of 25-mer oligonucleotide probes. These probe quartets consist of both perfect match (PM) and mismatch (MM, probes containing a single non-complementary base offset from the SNP interrogation position in the up or downstream direction) conformations for the major (A) and minor (B) SNP alleles being investigated. SNP genotype calls are ultimately made using the integration of fluorescent signal intensities at each location across the quartets.

To make each individual SNP genotype call the Affymetrix software employs a key mathematical filter, a feature extraction calculation, and finally fits each SNP into a trained statistical model. We will briefly review the Affymetrix calling approach on Affymetrix 10K Mapping Array. A more detailed description is available through Affymetrix or through previous publications [8]. The mathematical filter is termed the detection filter, which essentially determines if the MM fluorescence signal is greater than the PM signal. Such a result indicates a general inability of the tiled oligonucleotides to resolve the SNP from the background of mismatches whose sequences are nearly identical. SNPs that pass the detection filter are further utilized for feature extraction. It is during this calculation that the fluorescent signal intensities at each location on the microarray are indexed to calculate relative allele signal (RAS) values. Two RAS values are calculated for each SNP, one using the sense (RAS1) probes and a second using the antisense (RAS2) probes. The basic equation for RAS is as follows: RAS = A/(A+B), in which A represents the relative fluorescence intensities at the PM spots for the major SNP allele subtracted from the MM spots while B represents the same values for the minor allele. When plotted, the RAS1 and RAS2 values are used to infer a genotype call. For example, if a SNP has RAS1 and RAS2 values near 0,0 then the genotype call should be BB. If the RAS values are near 1,1 the genotype is AA. Unfortunately, the RAS values and the acceptable variance in each must be determined empirically for each SNP. Affymetrix genotyped 108 ethnically diverse DNA samples and utilized the corresponding RAS scores in a modified partitioning around medoids (MPAM) classification algorithm to delimit the boundaries of call silhouettes or zones for each SNP [8]. These call silhouettes are essentially statistical models for each SNP genotype based on the classification results of the training data set. They are used to make future experimental genotype calls. For further in-depth description of how calls are made on the 10K GeneChip^® ^array, see the manuscript by Liu *et al*. [9]. If a SNP's probe intensity values do not pass the detection filter score (DS) or the RAS scores fall outside the boundaries of the statistical model then the SNP is assigned a "NoCall" value. The overall call rate of a sample is equal to the number of SNPs receiving an AA, AB, or BB genotype call divided by the total number of SNPs on the chip.

After completing thousands of 10K GeneChip^® ^assays it is clear that even in samples with the highest overall call rates there are some SNPs consistently called less than other SNPs. In this article we report that infrequently called SNPs on the GeneChip^® ^Mapping 10K 2.0 Array are primarily due to problems associated with the boundaries of the statistical model call zone and therefore are related to suboptimal training of the MPAM algorithm for those particular SNPs. We detail the creation of an application, SNiPer, which utilizes two training-based clustering algorithms to increase overall call rates thereby increasing the amount of usable genotype data on each chip.

## Results

### Identification and characterization of poorly behaving SNPs on the 10K GeneChip^®^

**Figure 1:**
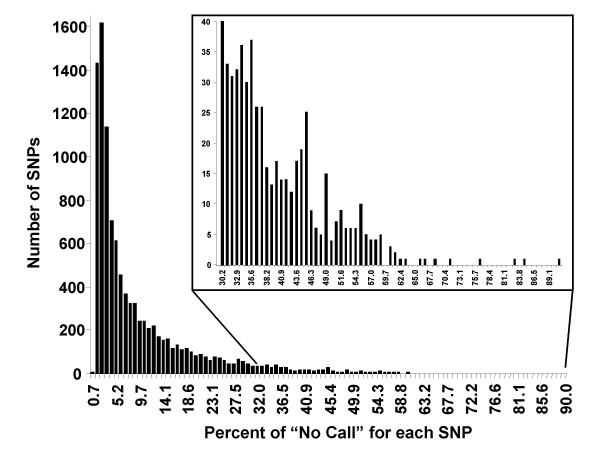
**Percentage of "NoCall" for SNPs on the 10K GeneChip**. SNP performance was investigated for 948 individual genotypes on the 10K GeneChip^® ^Mapping Array. SNPs were grouped based on their overall percentage of "No Call" signals.

**Figure 2:**
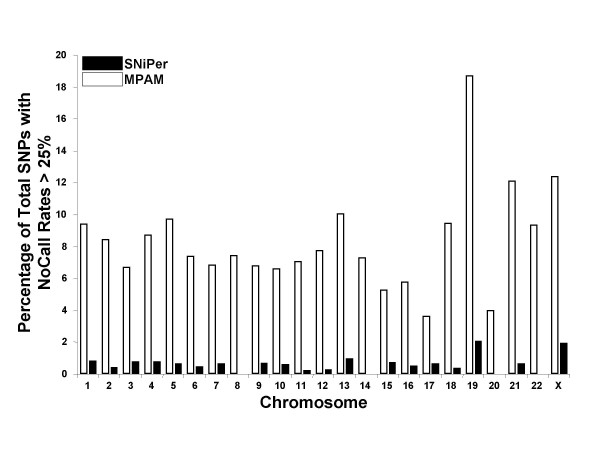
**Percentage of SNPs by chromosome with "No Call" rates greater than 25%**. SNPs having "No Call" rates greater than 25% were identified after processing with the MPAM (white bars) or SNiPer (black bars) algorithms. The total number of these poor performing SNPs was then divided by the total number of SNPs on the respective chromosome. The worst performing chromosome was 19 which is also known to have the highest gene density.

In order to identify those SNPs that frequently result in a "NoCall" on the 10K GeneChip^® ^we compiled a database of 948 individuals that were genotyped in the last two months in our laboratory. The call rate of these samples was required to be greater than 90%. The frequency at which each SNP was not called – the "NoCall" rate – was calculated (Figure 1). SNP identifiers and their observed "NoCall" rates are included as Additional File 1 and can be downloaded directly from our supplementary data site [10]. An arbitrary "NoCall" rate of 25% across the entire sample set was used to identify SNPs considered to be poor performers. The percentage of poorly performing SNPs on each chromosome as determined by the Affymetrix MPAM algorithm and the SNiPer algorithm are detailed in Figure 2.

**Figure 3:**
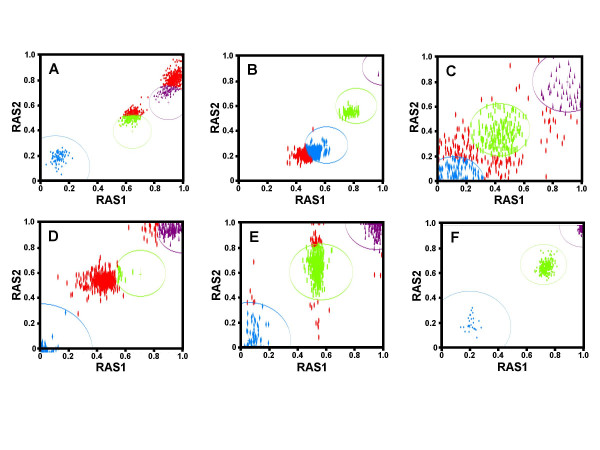
**A graphical representation of the performance of 6 example SNPs for 948 individuals**. Screen shots of the call zones (ellipses) and respective calls (solid shapes) for select SNPs from 948 individual genotypes. Blue represents call zone and calls of "B/B", Green represents "A/B", and Purple represents "A/A". Red represents those individuals that produced a "No Call" for the SNP. RAS1 and RAS2 scores are indicated on the x and y-axis respectively. Panel (**a**) SNP_A-1517236 and (**b**) SNP_A-1510986 represent SNPs with tightly clustered RAS scores, but inadequately trained call zones. An infrequently called SNP (SNP_A-1606312) with no systematic explanation is illustrated in panel **c**. Some SNPs cluster tightly at their RAS2 values, but have widespread RAS1 values (SNP_A-1513739) as in panel **d**. The opposite effect is seen in panel **e **(SNP_A-1508518). Panel **f **shows a SNP that is called >99% of the time (SNP_A-1511517) in these 948 individuals.

To investigate why certain SNPs behave poorly we examined four parameters: Detection filter scores (DS), G-C content of the tiled probe, PCR amplicon size, and the distribution of calls for each SNP in relation to the statistical model call zone. Comparison of the DS values clearly indicated that when well-performing SNPs (i.e. those with low "NoCall" rates) fail to be called they do so primarily because of the detection filter, while the majority of poorly performing SNPs fail for other reasons. For SNPs with "NoCall" rates less than 25%, the average "NoCall" rate was determined to be 5.5% ± 4.7% and the detection filter failure rate (the number of times across all 948 samples that the SNP fails the detection filter) was 2.9% ± 1.6%. Alternatively, for SNPs with "NoCall" rates greater than 25%, the average NoCall rate was 35.8% ± 11.6% and the detection filter failure rate was only 9.4% ± 10.3%. Failure of the detection filter causes ~50% of the total failures for the top performing SNPs but only ~25% of the total failures for the worst performing SNPs. Probe G-C content was not found to impact call rate. Interestingly, PCR amplicon size does play a role in the frequency at which a SNP is called. The Affymetrix specified PCR cycling parameters favor the production of amplicons less than 1 kb. The average amplicon size for the top 100 worst performing SNPs was 696 bp ± 181 bp while the 100 best performing SNPs were found on amplicons of 521 bp ± 87 bp (two-tailed t-test = p < 0.01). This finding underscores the fact that degraded sample DNA will result in lowered call rates, especially for those SNPs residing on larger sized amplicons. However, the samples used in our study consisted primarily of genomic DNA of high quality as determined by agarose gel electrophoresis. Therefore, while amplicon size can be linked to call rate, further investigation yielded that the more critical factor is the location of the MPAM model silhouette for each SNP. As indicated above, SNP failure of the detection filter is not the primary reason that the worst performing SNPs are not called. As an example one can look at the twenty worst performers. Only six of these SNPs fail the Affymetrix detection filter in at least one-third of the samples. Visual inspection of the GDAS call zones for the remaining SNPs suggests that the majority of the other poor performers are due to inadequate localization of the particular SNP model silhouette, a probable result of inadequate training of the Affymetrix MPAM algorithm for these SNPs. In other words, the RAS1/RAS2 intersection point was closely clustered for the SNP allele but still resulted in a "NoCall" because this cluster was primarily located outside the boundary of the silhouette. We were also able to find examples of widely varying RAS1 values in conjunction with tightly clustered RAS2 values and the opposite case as well. These findings are illustrated in Figure 3.

### SNiPer as a tool to call poorly performing SNPs

The ability to call these poorly performing SNPs was investigated using the algorithms discussed in the methods section. Through the use of real-time clustering we were able to decrease the average overall "NoCall" rate from 5.22% ± 0.03% to 0.97% ± 1.27% (Table 1). This was achieved by maintaining a 98.61% ± 0.21% genotype concordance compared to the Affymetrix genotypes (Table 1). Mendelian inheritance error was assessed using individually genotyped trios and was found to be comparable to the MPAM accuracy (99.94% for MPAM vs. 99.80% for SNiPer, Table 1).

**Table 1:** Comparison of the Affymetrix MPAM and SNiPer algorithms.

**ALGORITHM**	**%NOCALLS**	**CONCORDANCEVS. MPAM**	**INHERITANCE ACCURACY**
**MPAM**	5.22% ± 0.03%	------	99.94%
**SNiPer**	0.97% ± 1.27%	98.61% ± 0.21%	99.80%

## Discussion

In this article we identified 822 SNPs with "NoCall" rates of 25% or greater on the GeneChip^® ^10K Mapping Array. Additionally, we report the application of clustering algorithms to call these poorly performing SNPs at an increased rate without significantly compromising the concordance.

In regard to linkage studies on the 10K GeneChip^®^, the consequences of accurately adding 10% of SNPs which were previously not calculated include improved information content and filling gaps in the genetic map. As Figure 2 illustrates, the MPAM algorithm poorly calls over 18% of SNPs on chromosome 19. In fact, there are two stretches of SNPs on chromosome 19 where 5 out of 10 adjacent SNPs are poor performers. Additionally, chromosome 19 has the highest gene density of all human chromosomes, more than double the average for all other chromosomes [11]. It is unfortunate that this chromosome contains the lowest density of SNPs of all the autosomes on the 10K GeneChip^® ^platform. Importantly, only 2% of the SNPs on chromosome 19 exhibit "NoCall" rates greater than 25% after running the samples through the SNiPer algorithm.

There are 12 regions in the genome where three consecutive SNPs exhibit "NoCall" rates greater than 25% of the time, three of these regions occurring on chromosome 1. Processing of samples using the SNiPer algorithm resolves this issue. After running SNiPer the highest number of poorly performing neighboring SNPs is 2 in a window size of 10 and there now exists no regions in the genome with consecutive SNPs with "NoCall" rates greater than 25%.

Interestingly, it appears that SNPs can fail the MPAM calling algorithm in four different ways. A widely dispersed RAS1 (Figure 3d) or RAS2 (Figure 3e) value can lead to a poorly performing SNP. Tightly clustered RAS1 and RAS2 values complemented with an inadequately trained call zone (Figure 3a,b) are the more frequent reason a SNP performs poorly. Also, a small percentage of SNPs fail to elicit clustered RAS scores for no clear systematic reason (Figure 3c).

Even though the genomics community is moving towards denser SNP genotyping platforms for both linkage and association analysis there are still a large number of funded studies currently being performed using the 10K GeneChip. For this reason it still remains important to improve upon the performance of the assay whenever possible. Additionally, even though the SNiPer algorithms detailed in this manuscript were designed for use on the 10K GeneChip^® ^it could be applied to the denser genotype platforms from Affymetrix with little modification. One future direction of study may include the comparison of the SNiPer algorithm with the dynamic modeling algorithm currently in use on the 100K and 500K GeneChips.

## Conclusion

SNPs called less than 75% of the time occur at a frequency of 8% on the GeneChip^® ^10K Mapping Array. While there is a relationship between frequency of calling and PCR amplicon size we have concluded that the primary reason for a high "NoCall" rate is inadequate training of the calling algorithm. These poorly performing SNPs could play a confounding role in linkage analysis studies especially on chromosomes 19, 21, and X, where the proportion of poorly performing SNPs is greater than 10% of the total interrogated SNPs on the entire chromosome. The SNiPer algorithms now successfully call these poorly performing SNPs, resulting in increased performance of the 10K GeneChip.

## Methods

### 10K GeneChip^® ^Mapping Array Genotyping

10K SNP genotyping was performed as detailed by Affymetrix on the GeneChip^® ^Mapping 10K 2.0 Array [12]. In short, 250 ng of genomic DNA was digested with 10 units of Xba I (New England Biolabs, Beverly, MA) for 2 hours at 37°C. Adaptor Xba (P/N 900410, Affymetrix, Santa Clara, CA) was then ligated onto the digested ends with T4 DNA Ligase for 2 hours at 16°C. After dilution with water, samples were subjected to PCR using primers specific to the adaptor sequence (P/N 900409, Affymetrix) with the following amplification parameters: 95°C for 3 minutes initial denaturation, 95°C 20 seconds, 59°C 15 seconds, 72°C 15 seconds for a total of 35 cycles, followed by 72°C for 7 minutes final extension. PCR products were then purified and fragmented using 0.24 units of DNase I at 37°C for 30 minutes. The fragmented DNA was then end-labeled with biotin using 100 units of terminal deoxynucleotidyl transferase at 37°C for 2 hours. Labeled DNA was then hybridized onto the 10K Mapping Array at 48°C for 16–18 hours at 60 rpm. The hybridized array was washed, stained, and scanned according to the manufacturer's instructions.

### SNiPer

The SNiPer program was implemented in Java using Sun Microsystem's free Java 2 Standard Edition 5.0 (J2SE 5.0) compiler [13]. The user interface was constructed using Swing and Abstract Windowing Toolkit components; both standard class libraries provided in the Java Foundation Classes as part of J2SE 5.0. Java was chosen for the portability of the Java Virtual Machine.

SNiPer was created to increase call rates without sacrificing accuracy. Two main approaches were explored; the creation of new static models based on our large database of individual genotypes or the development of a way to cluster new samples against an existing library of data. The second option was investigated further because it affords the end user the ability to adapt the clustering as new data is generated much more easily. However there are two major problems facing real-time clustering. The first is prohibitively long runtimes and the second is the elucidation of the proper input parameters for the algorithm variables. The runtime issue can be solved by proper algorithm choice and optimization of the algorithm for increased efficiency. The second hurdle is relatively straightforward for individual genotyping purposes due to the knowledge that the data should cluster in three separate groups.

Algorithm choice began with the investigation of PAM, CLARANS, and WAVECLUSTER. PAM and CLARANS are both medoid-based partitioning algorithms and both were found to produce high quality clusters. However, they were abandoned because of extremely poor runtime efficiency on large data sets that make real-time clustering time-consuming. WAVECLUSTER is a wavelet transformation algorithm known to scale extremely well to very large data sets because it requires only one pass through the data. We focused on the sequential use of two algorithms known as PANN (Partitioning Around Nearest Neighbors) and MDBSCAN (Modified Density Based Spatial Clustering of Applications with Noise) since they are less sensitive to input parameters.

PANN is a partitioning algorithm similar to K-Means except it utilizes the Affymetrix distance between groups correction in place of the typical distance to the nearest centroid calculation. K-Means clustering fails because it tends to split high-density clusters while PANN takes advantage of the fact that the number of clusters and their approximate locations can be predicted. PANN uses a naïve approach for its initial assignment and reassigns points with a correction based upon the ideal that a point should belong to the cluster with the nearest neighbor. The steps of PANN can be summarized as following:

1. Calculate the three centroids representative of the three clusters.



2. Assign each point to the cluster with the nearest centroid.

3. For each point, find its nearest neighbor in each of the three clusters. Assign each point to the cluster with the smallest nearest neighbor distance.

4. Repeat Step 3 until results converge (i.e. no points are moved to different clusters).

MDBSCAN is a modified version of the DBSCAN algorithm that includes a pre-processing filter for calculating input parameters and a post-processing filter to assign points considered noise [14]. MDBSCAN is designed to discover a variable number of clusters, which allows it to easily discover and avoid calling SNPs which do not have three clear clusters. The steps of MDBSCAN can be summarized as following:

1. Calculate the value epsilon, . Experiments determined that results converged for the value λ = 35.

2. For each point, find the epsilon neighborhood *N*_*Eps*_, the set of all points that are within *Eps *distance from the current point.

3. For each point, if *size*(*N*_*Eps*_) ≥ *MinPts*, then mark it as a core point. For our purposes the value *MinPts *= 4 was used.

4. Find a random core point and add it to a new cluster.

5. For each core point in the cluster, add all the points in its *N*_*Eps *_to the cluster and remove them from the database.

6. Repeat Step 5 until no more new points can be added to the cluster.

7. Repeat Step 4 until no more core points remain in the database.

8. For the remaining points in the database, assign them to the cluster with the nearest centroid.

Our investigations found that the best performance is derived through the sequential use of the PANN and MDBSCAN algorithms. The input data required by these algorithms is the same; columns containing Affymetrix SNP ID numbers, predetermined (via Affymetrix GDAS) or dummy genotype calls, and columns denoting the RAS scores in both the antisense and sense direction for each SNP. A file is then designated as the data output location and the data is clustered using both algorithms. If a SNP does not pass the Affymetrix DS threshold then it also receives a "NoCall" from SNiPer and is not clustered. SNiPer is designed to handle multiple samples at once and we have successfully clustered and called 96 samples in ~60 minutes time. After generating a data set from each algorithm a "strict" filter is applied whereby if the genotype calls did not agree between PANN and MDBSCAN the final output for that SNP was a "NoCall". SNiPer can be downloaded freely from the supplementary data page [10].

### PCR amplicon script

Amplicon sizes were determined by taking the chromosomal location of each SNP on the microarray chip and finding the nearest upstream and downstream cut sites for the Xba nuclease. The SNP chromosomal locations were extracted from chromosome report data files downloaded from NCBI's FTP site [15]. Xba cut sites were determined by software, developed in-house in Perl, that processed chromosomal FASTA sequence files downloaded from UCSC's Genome Browser FTP site [16].

## List of abbreviations used

SNP: single nucleotide polymorphism

PCR: polymerase chain reaction

FSP: fragment selection by PCR

RAS: relative allele signal

## Authors' contributions

MJH performed SNP genotyping, participated in the conceptualization of SNiPer, and drafted the manuscript. DWC helped conceptualize and write code for SNiPer, performed the statistical analysis of the SNP data, and helped draft the manuscript. ADS helped conceptualize and write code for SNiPer. JJC performed analysis of SNiPer output data. DH-L performed SNP genotyping. JVP performed statistical analysis of the SNP data and implemented the PCR amplicon script. DAS provided oversight and funding for the project. All of the authors have read and approved the final manuscript.

## Supplementary Material

Additional File 1 SNP identifiers and their observed No Call Rates. Click here for file
